# Treatment with didemnin B, an elongation factor 1A inhibitor, improves hepatic lipotoxicity in obese mice

**DOI:** 10.14814/phy2.12963

**Published:** 2016-09-13

**Authors:** Alexandra M. Hetherington, Cynthia G. Sawyez, Brian G. Sutherland, Debra L. Robson, Rigya Arya, Karen Kelly, René L. Jacobs, Nica M. Borradaile

**Affiliations:** ^1^ Department of Physiology and Pharmacology Schulich School of Medicine and Dentistry Western University London Ontario Canada; ^2^ Robarts Research Institute Schulich School of Medicine and Dentistry Western University London Ontario Canada; ^3^ Department of Medicine Schulich School of Medicine and Dentistry Western University London Ontario Canada; ^4^ Metabolic and Cardiovascular Diseases Laboratory Department of Agricultural, Food and Nutritional Science University of Alberta Edmonton Alberta Canada

**Keywords:** ER stress, lipids, lipotoxicity, nonalcoholic fatty liver disease, obesity

## Abstract

Eukaryotic elongation factor EEF1A1 is induced by oxidative and ER stress, and contributes to subsequent cell death in many cell types, including hepatocytes. We recently showed that blocking the protein synthesis activity of EEF1A1 with the peptide inhibitor, didemnin B, decreases saturated fatty acid overload‐induced cell death in HepG2 cells. In light of this and other recent work suggesting that limiting protein synthesis may be beneficial in treating ER stress‐related disease, we hypothesized that acute intervention with didemnin B would decrease hepatic ER stress and lipotoxicity in obese mice with nonalcoholic fatty liver disease (NAFLD). Hyperphagic male *ob/ob* mice were fed semipurified diet for 4 weeks, and during week 5 received i.p. injections of didemnin B or vehicle on days 1, 4, and 7. Interestingly, we observed that administration of this compound modestly decreased food intake without evidence of illness or distress, and thus included an additional control group matched for food consumption with didemnin B‐treated animals. Treatment with didemnin B improved several characteristics of hepatic lipotoxicity to a greater extent than the effects of caloric restriction alone, including hepatic steatosis, and some hepatic markers of ER stress and inflammation (GRP78, *Xbp1s*, and *Mcp1*). Plasma lipid and lipoprotein profiles and histopathological measures of NAFLD, including lobular inflammation, and total NAFLD activity score were also improved by didemnin B. These data indicate that acute intervention with the EEF1A inhibitor, didemnin B, improves hepatic lipotoxicity in obese mice with NAFLD through mechanisms not entirely dependent on decreased food intake, suggesting a potential therapeutic strategy for this ER stress‐related disease.

## Introduction

ER stress has been linked to the pathogenesis and progression of metabolic diseases, including obesity, type 2 diabetes, and nonalcoholic fatty liver disease (NAFLD) (Lee and Ozcan 2014). Although various stimuli lead to ER stress in these diseases, the cellular responses to either restore homeostasis or to remove damaged cells are relatively well conserved. Initially, activation of PERK‐ and IRE1‐mediated pathways attenuates global protein translation to reduce the burden of protein processing within the ER. However, with prolonged stress, protein synthesis rates recover through an ATF4‐ and CHOP‐dependent mechanism, which re‐establishes the anabolic burden at the ER, leading to oxidative stress and cell death (Han et al. 2013; Krokowski et al. 2013). Thus, it has been proposed that inhibiting protein synthesis may be a therapeutic strategy for treating ER stress‐related diseases (Han et al. 2013). In support of this concept, we recently showed that decreasing protein synthesis with an inhibitor of eukaryotic elongation factor 1A (EEF1A), didemnin B, protected HepG2 cells from death induced by exposure to high palmitate (Stoianov et al. 2015), an in vitro model of the hepatocyte lipotoxicity that occurs during NAFLD progression.

EEF1A was originally identified as the mediator of GTP‐dependent recruitment of aa‐tRNA to the ribosome (McKeehan and Hardesty 1969). Mammals express two isoforms of this protein (designated 1 and 2), which share 92% identity of amino acid sequence, are generally mutually exclusively expressed in mouse and human tissues, and function similarly with regard to their role in peptide elongation (Mateyak and Kinzy 2010). EEF1A1 is the only isoform expressed in liver (Newbery et al. 2007). EEF1A has also been implicated in the regulation of diverse cellular processes, including cytoskeleton dynamics, protein degradation, and cell death – functions which may or may not be shared by both isoforms (Mateyak and Kinzy 2010). In secretory cell types, including hepatocytes and pancreatic *β*‐cells, EEF1A1, in particular, appears to be highly localized to the ER (Chen et al. 2010; Stoianov et al. 2015). Furthermore, we and others have shown that EEF1A1 is highly expressed in adipose and steatotic tissues (including liver) (Borradaile et al. 2006; Lee et al. 2009; Buque et al. 2010; Stoianov et al. 2015), that it is induced in response to ER stress and oxidative stress, and that it promotes cell death under these conditions (Chen et al. 2000; Borradaile et al. 2006).

The isolation and identification of didemnin B as a selective inhibitor of EEF1A peptide elongation activity has provided a valuable tool for studies of this aspect of EEF1A1 function. Didemnin B is a cyclic depsipeptide produced by marine tunicates that specifically binds the GTP‐bound conformation of EEF1A, inhibiting its release from the ribosomal A site and preventing subsequent peptide elongation (Marco et al. 2004). This compound was the first marine organism‐derived drug to undergo clinical trials, as a potential anticancer, antiviral, and immunosuppressive agent (Lee et al. 2012). These trials were initiated and ultimately halted before the discovery of EEF1A as the binding target for didemnin B, which is thought to be the mechanism responsible for its diverse biological effects. We previously identified EEF1A1 as a mediator of fatty acid‐induced (lipotoxic) cell death in several cell types, including hepatocytes, and found that it participates in this process downstream of ER stress (Borradaile et al. 2006; Stoianov et al. 2015). Moreover, using didemnin B, we determined that the protein synthetic function of EEF1A1 is responsible for the progression from ER stress to cell death in hepatocytes (Stoianov et al. 2015). Neither inhibition of EEF1A1 nor inhibition of protein synthesis has been tested as possible interventions for ER stress‐related metabolic disease in vivo. Thus, we investigated whether acute treatment with the EEF1A inhibitor, didemnin B, could improve hepatic ER stress and lipotoxicity, and liver pathology and function in leptin‐deficient *ob/ob* mice. This hyperphagic genetic model of early NAFLD exhibits severe hepatic steatosis, mild lobular inflammation, and hepatic lipotoxicity, in the setting of obesity.

## Materials and Methods

### Mice

Five‐week‐old male C57BL/6J and leptin‐deficient (*ob/ob*) mice on a C57BL/6J background (Jackson Laboratory, Bar Harbor, ME) were fed ad libitum on AIN‐76A semipurified diet (17.7% protein, 64.9% carbohydrate, and 5.2% fat by weight [Harlan Teklad, Toronto, ON, Canada]) for 4 weeks. All studies were approved by the Western University Council on Animal Care. During week 5, mice were given intraperitoneal (i.p.) injections of didemnin B (50 *μ*g/kg) or vehicle control on days 1, 4, and 7. The dose and treatment regimen were chosen based on previous work indicating that 50 *μ*g/kg was sufficient to partially inhibit protein synthesis in solid tumors in mice (Robert et al. 2009), and that the half‐life of didemnin B in liver is 78 h (Beasley et al. 2005). Didemnin B was obtained from the Open Chemical Repository of the Developmental Therapeutics Program at the National Cancer Institute, and solubilized in 1% dimethyl sulfoxide, 5.2% PEG400, 5.2% Tween 80, and 88.6% sterile saline (Robert et al. 2009). Mice were individually caged and body weights and food consumption were measured daily. A group of *ob/ob* plus vehicle control mice were pair‐fed to match *ob/ob* plus didemnin B mice for caloric intake. Prior to sacrifice, glucose tolerance was measured following a 6‐h fast and oral gavage of a 20% solution of d‐glucose (BDH Chemicals, Mississauga, ON, Canada) to deliver 1 g of glucose per kg body weight. Insulin tolerance was measured following a 6‐h fast and i.p. injection of 0.6 IU of insulin (Novo Nordisk, Mississauga, ON, Canada) per kg body weight. Body weight, epididymal fat weight, and liver weight were determined at sacrifice. Blood glucose was determined by glucometer (Bayer Healthcare, Mississauga, ON, Canada). Plasma insulin was measured by ultrasensitive ELISA (Alpco Diagnostics, Salem, NH).

### Liver lipids

Total liver lipids were extracted by the Folch method (Folch et al. 1957) from samples frozen in liquid nitrogen and subsequently stored at −80°C. Triglycerides, free cholesterol, and total cholesterol from chloroform extracts of liver tissue were determined by enzymatic, colorimetric assays (triglyceride, Roche Diagnostics (Indianapolis, IN); cholesterol, Wako Diagnostics, Richmond, VA) (Assini et al. 2013). All plasma and liver lipid measurements were performed through the Metabolic Phenotyping Laboratory in Robarts Research Institute.

### Histology

Liver and pancreatic samples were embedded in optimal cutting temperature compound (Sakura Finetek USA, Inc., Torrance, CA) at the time of sacrifice. Hepatic sections (8 *μ*m) were prepared with a cryostat (Leica Biosystems, Concord, ON, Canada) and stained with either hematoxylin and eosin (H&E) (Leica Biosystems) or Oil Red O (Sigma, St. Louis, MO). Sections were imaged using an Olympus BX51 microscope (Center Valley, PA). Severity of NAFLD was quantified using the NAFLD Activity Score (NAS) developed by the Pathology Committee of the Non‐Alcoholic Steatohepatitis Research Network (Kleiner et al. 2005), which includes analysis of steatosis, lobular inflammation, and hepatocellular ballooning. Steatosis was evaluated at 10× magnification, and inflammation and ballooning were evaluated at 20× magnification, in three random fields of view per mouse. Steatosis was scored from 0 to 3, with a score of 0 = <5% of section area covered by steatosis, 1 = 5–33%, 2 = 33–66%, and 3 = >66%. Lobular inflammation was scored from 0 to 3, with a score of 0 representing no inflammatory foci present per section area, 1 representing <2 foci present, 2 representing 2–4 foci present, and 3 representing >4 foci present. Ballooning was scored from 0 to 2, with 0 representing no ballooned cells present per section area, 1 representing few ballooned cells, and 2 representing prominent ballooning. Average steatosis, lobular inflammation, and ballooning score per mouse were calculated and an average NAS ranging from 0 to 8 was determined.

Pancreatic sections (8 *μ*m) were prepared with a cryostat (Leica Biosystems) and stained for insulin using a rabbit polyclonal antibody (Cell Signaling Technology, Danvers, MA) followed by HRP‐conjugated polyclonal anti‐rabbit secondary antibody (Santa Cruz Biotechnology, Dallas, TX), and visualized using diaminobenzidine (Vector Laboratories, Burlington, ON, Canada). Sections were imaged using an Olympus BX51 microscope at 10× magnification in three random fields of view per mouse. Total islet area per section area and average islet density per mouse were evaluated using ImageJ (NIH, Bethesda, MD). Pancreatic islets were categorized as small (<2500 *μ*m^2^), medium (2500 *μ*m^2^ ≤ *x* ≤ 10,000 *μ*m^2^), or large (>10,000 *μ*m^2^) to determine islet size distribution (Han et al. 2015).

### Plasma liver enzymes

Plasma alanine aminotransferase (ALT) and aspartate aminotransferase (AST) were measured by enzymatic rate assays (Roche Diagnostics) using an autoanalyzer at the London Health Sciences Centre Core Laboratory.

### Immunoblotting

Tissue lysates were prepared using RIPA buffer containing protease and phosphatase inhibitors. Total protein was quantified using the Pierce Bicinchoninic Acid Protein Assay Kit (Thermo Scientific, Mississauga, ON, Canada). Tissue lysates of 75–100 *μ*g were resolved under reducing conditions by 10% SDS‐PAGE and transferred to polyvinyl difluoride membrane. EEF1A1, GRP78, phosphorylated (phospho) JNK, JNK, phospho eIF2*α*, eIF2*α* (Cell Signaling Technology), albumin (Thermo Scientific), and actin (Sigma) were detected with rabbit polyclonal antibodies and an HRP‐conjugated polyclonal anti‐rabbit secondary antibody (Santa Cruz Biotechnology). Bands were visualized by chemiluminescence, and those corresponding to EEF1A1, GRP78, phospho eIF2*α*, eIF2*α*, albumin, and actin ran at approximately 50, 78, 38, 65, and 42 kDa, respectively. Bands corresponding to phospho JNK and JNK ran as a doublet at 46 and 54 kDa. Bands were quantified using Quantity One (BioRad, Mississauga, ON, Canada).

### Gene expression analysis

Expression of a panel of genes relevant to metabolism, cellular stress, and inflammation was analyzed as described previously (da Silva et al. 2014). Total RNA was isolated from frozen liver tissues using Trizol (Invitrogen, Burlington, ON, Canada). RNA quality was assessed with an Agilent 2100 bioanalyzer, using an RNA 6000 Nano kit (Agilent Technologies, Santa Clara, CA), followed by reverse transcription using Superscript II (Invitrogen). Primer sets and a corresponding probe for each gene of interest were designed using the Universal Probe Library (Roche Diagnostics) based on the NCBI reference nucleotide sequences for *Mus musculus*. Each primer pair and probe combination was tested by qPCR (StepOnePlus, Applied Biosystems, Mississauga, ON, Canada) to confirm that amplification conditions were suitable for use in the Biomark gene chip (Fluidigm, Markham, ON, Canada). A mix containing primers for all genes combined in a single assay was used to preamplify the cDNA in each sample. Preamplification was performed in order to enrich the template cDNA for use in the Biomark gene chip. All preamplified samples were tested on the StepOnePlus qPCR machine using the probe for cyclophilin (*Ppia*) before loading of the Biomark gene chip. Primer pairs and preamplified samples were loaded into separate wells on a 96‐by‐96 gene chip (Fluidigm). qPCR was run on the Biomark system (Fluidigm) for 40 cycles. Relative RNA expression for each gene in a sample was calculated using the comparative threshold (ΔΔCT) method. Values were normalized to the endogenous housekeeping gene *Ppia*. All samples were assayed in triplicate for each primer pair.

### Plasma lipids and fast performance liquid chromatography

Plasma triglyceride, total cholesterol, and free fatty acid concentrations were determined by spectrophotometric assays (Roche Diagnostics). Plasma apolipoprotein A‐1 concentrations were measured using a mouse‐specific ELISA (Cloud‐Clone, Houston, TX). Plasma lipoprotein distribution was determined by fast performance liquid chromatography (FPLC) (Assini et al. 2013). Fresh EDTA plasma (50 *μ*L) was separated by FPLC using an AKTA purifier (GE Healthcare Life Sciences, Mississauga, ON, Canada) and a Superose 6 column (GE Healthcare Life Sciences). A constant flow rate of 0.4 mL/min was used to collect 750 *μ*L fractions. A 125‐*μ*L aliquot of each fraction was used to measure total cholesterol and triglyceride enzymatically in samples on a 96‐well microtitre plate with 75 *μ*L of two times concentrated reagents (triglyceride, Roche Diagnostics; cholesterol, Wako Diagnostics; standards, Randox, Kearneysville, WV).

### Statistics

All calculations and analyses were performed using GraphPad Prism. One‐way ANOVA followed by post hoc tests, or Student's *t*‐tests were used to determine significant differences at *P* < 0.05. For ANOVA, significant differences between groups are indicated with different letters on data plots. Bars with different letters are significantly different at *P* < 0.05, while those that share the same letter are not significantly different. Lower case and capital letters indicate separate statistical analyses that are not comparable. In cases where means did not reach statistical significance by ANOVA, but changes were pronounced, *t*‐tests were performed with *P* values indicated on the corresponding data plots.

## Results

### Didemnin B treatment modestly reduces food consumption in obese mice

To determine whether chemical inhibition of EEF1A1 activity could improve hepatic lipotoxicity in a mouse model of obesity and metabolic syndrome, we used 5‐week‐old male C57BL/6J (lean control) and leptin‐deficient *ob/ob* mice. All mice were fed AIN‐76A diet for 4 weeks. During week 5, mice were given i.p. injections of didemnin B (50 *μ*g/kg) or vehicle control on days 1, 4, and 7. Interestingly, we found that administration of didemnin B modestly decreased food intake by 22% without evidence of illness or distress. Therefore, we included an additional control group of vehicle‐treated animals matched for food consumption with didemnin B‐treated animals (calorie restricted) (Table 1). As expected, body weight and epididymal fat weight were significantly increased in all *ob/ob* animals compared to lean control animals (Table 1). Neither treatment with didemnin B nor caloric restriction affected body weight or epididymal fat weight over the course of the experiment (Table 1).

**Table 1:** Parameters of obesity and fatty liver in experimental mouse groups

Mouse strain	C57BL/6J	*ob/ob*	*ob/ob*	*ob/ob*
Treatment	Vehicle	Vehicle	Didemnin B	Calorie‐restricted vehicle
Number of mice	12	12	12	8
Food consumption (kcal/day)	8.9 ± 0.3 (a)	13.5 ± 0.4 (b)	10.5 ± 0.6 (a)	10.3 ± 0.5 (a)
Body weight (g)	23.7 ± 0.5 (a)	38.2 ± 1.7 (b)	38.3 ± 1.7 (b)	38.4 ± 0.9 (b)
Epididymal fat weight (g)	0.4 ± 0.0 (a)	2.6 ± 0.2 (b)	2.6 ± 0.1 (b)	2.8 ± 0.1 (b)
Liver weight (g)	1.0 ± 0.0 (a)	2.6 ± 0.2 (b)	2.0 ± 0.1 (c)	2.0 ± 0.1 (c)
Liver triglyceride (mg/g)	39.8 ± 7.9 (a)	223.5 ± 32.1 (b)	152.3 ± 10.7 (c)	159.4 ± 11.2 (bc)
Liver cholesteryl ester (mg/g)	1.3 ± 0.2 (a)	6.0 ± 0.6 (b)	8.7 ± 1.5 (c)	4.5 ± 0.7 (b)
Liver free cholesterol (mg/g)	2.1 ± 0.1 (a)	3.8 ± 0.4 (b)	3.1 ± 0.2 (ab)	3.7 ± 0.4 (b)

Five‐week‐old C57BL/6J and *ob/ob* mice were maintained ad libitum or pair‐fed (*ob/ob* + calorie restricted) on AIN‐76A semipurified diet for 4 weeks. During week 5, mice received i.p. injections of either vehicle or didemnin B (50 *μ*g/kg), as indicated, on days 1, 4, and 7. Upon sacrifice body weights, epididymal fat weights, and liver weights were determined, and tissues were harvested and frozen in liquid nitrogen. Liver triglyceride, cholesteryl ester, and free cholesterol concentrations were measured in Folch extracts using standard enzymatic, colorimetric assays. Data are means ± SEM, *n* = 8–12. Statistically significant differences were determined by ANOVA followed by post hoc tests comparing all groups. Values with different letters are significantly different at *P* < 0.05, while those that share the same letter are not significantly different.

### Didemnin B treatment reduces hepatic lipid content and improves NAFLD histopathology in obese mice

Liver weights were decreased by 23% in both didemnin B and calorie‐restricted animals, compared to *ob/ob* vehicle control mice (Table 1). These changes in liver weight were associated with significant changes in liver lipid contents which, with the notable exception of cholesteryl esters, were similar for didemnin B‐treated and calorie‐restricted mice. Liver triglyceride levels were decreased by 32%, while liver cholesteryl ester levels were increased by 45% in didemnin B‐treated mice compared to *ob/ob* vehicle control mice (Table 1). No differences in liver free cholesterol levels were observed among *ob/ob* vehicle control and treatment groups (Table 1).

**Figure 1:**
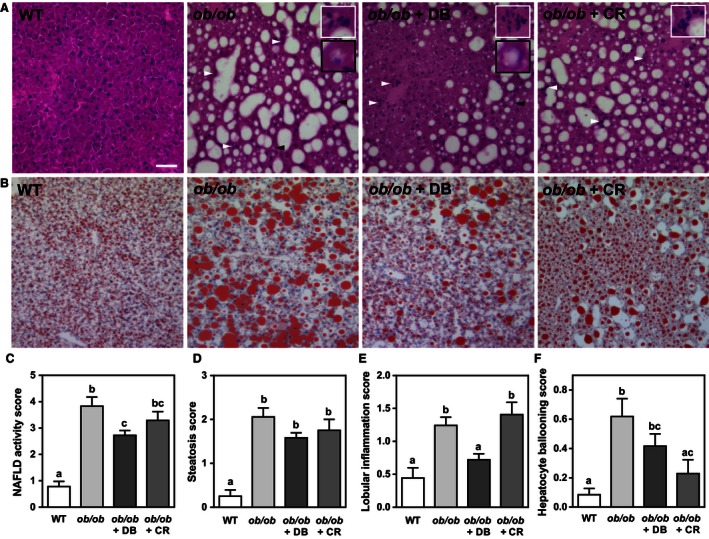
Didemnin B treatment improves nonalcoholic fatty liver disease (NAFLD) histopathology in obese mice. Five‐week‐old C57BL/6J (WT) and obese (*ob/ob*) mice were maintained ad libitum on semipurified diet for 4 weeks. During week 5, mice received i.p. injections of either vehicle or didemnin B (50 *μ*g/kg), as indicated, on days 1, 4, and 7. An additional group of *ob/ob* mice was pair‐fed to match reduced caloric intake of didemnin B‐treated mice (calorie restricted, CR). Upon sacrifice, liver tissues from WT,* ob/ob*,* ob/ob* + didemnin B (DB), and *ob/ob* + CR mice were harvested, embedded in optimal cutting temperature, sectioned, and stained with either (A) H&E to visualize tissue morphology and inflammatory infiltrates or (B) Oil Red O to visualize lipid droplets. White arrows indicate inflammatory infiltrates and black arrows indicate ballooned hepatocytes. Higher magnification inset images show inflammatory infiltrates outlined in white and ballooned hepatocytes outlined in black. Scale bar represents 100 *μ*m. (C) NAFLD activity scores, (D) steatosis activity scores, (E) lobular inflammation scores, and (F) hepatocyte ballooning scores were assessed in three hepatic H&E‐stained serial sections per mouse. Data are means ± SEM,* n* = 8–12. Statistically significant differences were determined by ANOVA followed by post hoc tests comparing all groups. Bars with different letters are significantly different at *P* < 0.05, while those that share the same letter are not significantly different.

Gross morphology and lipid droplet content of hepatic tissues were assessed by H&E and Oil Red O staining, and light microscopy. As expected, liver sections from *ob/ob* mice showed dramatically increased lipid droplet size compared to sections from lean control mice. Liver sections from mice treated with didemnin B showed a reduction in the overall appearance of lipid droplets, which was similar to that observed in calorie‐restricted mice (Fig. 1A, B). The degree of steatosis, hepatocellular ballooning, lobular inflammation and the overall severity of NAFLD were graded histologically (Fig. 1C–F). Didemnin B treatment significantly reduced the presence of lobular inflammation by 42%, independent of caloric restriction (Fig. 1E), resulting in a 29% reduction in overall NAS (Fig. 1C) compared to *ob/ob* vehicle control‐treated animals. However, in calorie‐restricted animals, hepatocellular ballooning decreased by 63% compared to *ob/ob* control animals (Fig. 1F), an observation which did not reach statistical significance in didemnin B‐treated mice. We did not observe fibrosis in the livers of any of the mice in the four experimental groups.

### Didemnin B treatment improves markers of hepatic lipotoxicity in obese mice

**Figure 2:**
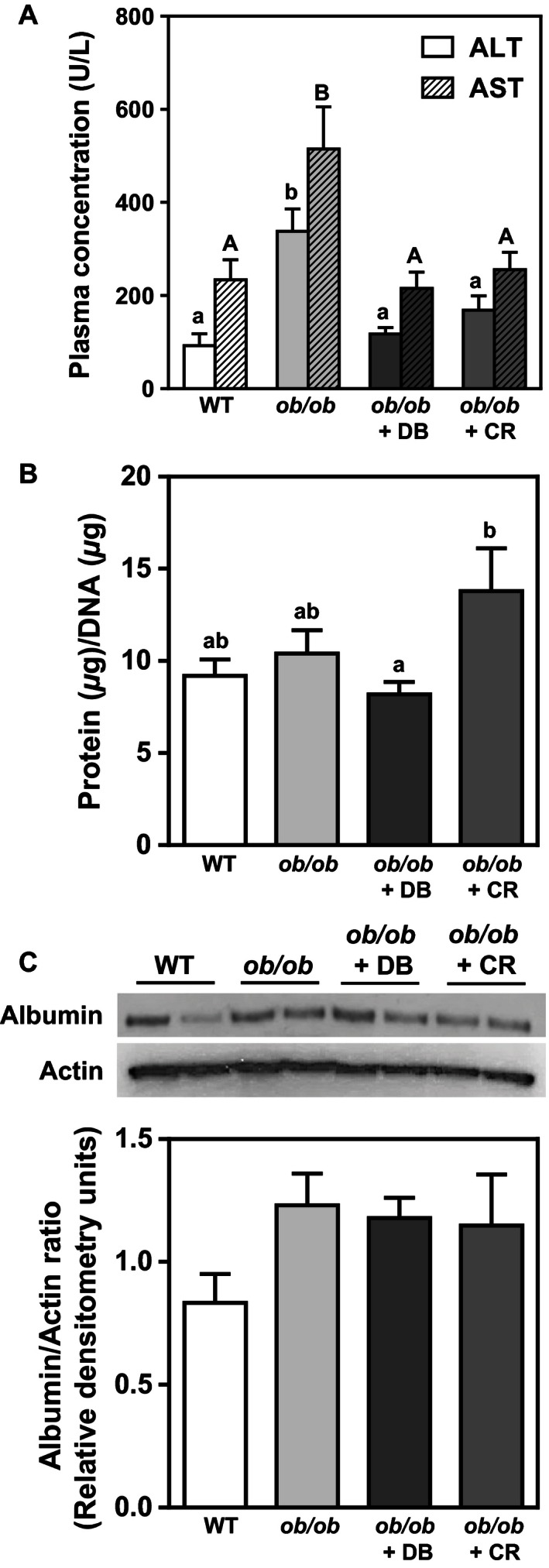
Both didemnin B treatment and caloric restriction improve obesity‐associated liver damage. C57BL/6J (WT) and obese (*ob/ob*) mice were treated as described in Figure 1. (A) Plasma ALT and AST were measured post mortem using enzymatic rate assays. (B) Hepatic protein contents (*μ*g) per DNA contents (*μ*g) were measured by BCA assay and NanoDrop, respectively. (C) Albumin protein was detected in whole tissue liver homogenates by immunoblotting and quantified by densitometry. Data are expressed relative to actin. Representative blots are shown. Data are means ± SEM,* n* = 8–12. Statistically significant differences were determined by ANOVA followed by post hoc tests comparing all groups. Bars with different letters are significantly different at *P* < 0.05, while those that share the same letter are not significantly different. Lower case and capital letters indicate separate statistical analyses that are not comparable.

Hepatic lipotoxicity in mouse models of NAFLD is associated with increased circulating levels of the liver enzymes ALT and AST, which escape to the circulation from damaged hepatocytes (Tsutsumi et al. 2011). Plasma ALT levels in didemnin B‐treated mice and calorie‐restricted mice were decreased by 65% and 50%, respectively, compared to *ob/ob* vehicle control mice. Similarly, plasma AST levels were decreased by 58% and 50% in didemnin B‐treated and calorie‐restricted animals, respectively, compared to *ob/ob* vehicle control mice (Fig. 2A). To further assess liver damage, total hepatic protein was measured in comparison to total hepatic DNA, and liver albumin contents were determined by immunoblotting. Hepatic protein:DNA ratio was increased by 69% in the livers of calorie‐restricted mice compared to didemnin B‐treated mice, but was not significantly different between any other groups (Fig. 2B). Similarly, liver albumin contents remained unchanged between experimental groups (Fig. 2C). These data suggest that didemnin B can improve overt NAFLD‐associated liver damage, and that this effect is partly due to reduced food intake in these mice. Moreover, the lack of significant reduction of steady‐state liver total protein and albumin contents with didemnin B (Fig. 2B, C) suggests that our treatment protocol (low dose and intermittent administration) is not hepatotoxic.

**Figure 3:**
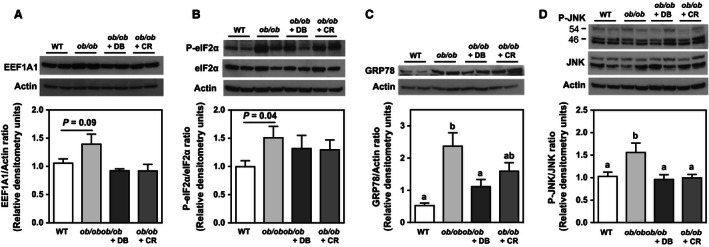
Didemnin B treatment improves protein markers of hepatic ER stress. C57BL/6J (WT) and obese (*ob/ob*) mice were treated as described in Figure 1. (A) EEF1A1, (B) phosphorylated eIF2*α* (P‐eIF2*α*) and total eIF2*α*, (C) GRP78, and (D) phosphorylated JNK (P‐JNK) and total JNK proteins were detected by immunoblotting in whole liver tissue homogenates. Representative blots are shown. Bands were quantified by densitometry, and relative densitometry units were expressed relative to actin or total corresponding protein. Data are means ± SEM,* n* = 8–12. Statistically significant differences were determined by ANOVA followed by post hoc tests comparing all groups. Bars with different letters are significantly different at *P* < 0.05, while those that share the same letter are not significantly different.

**Figure 4:**
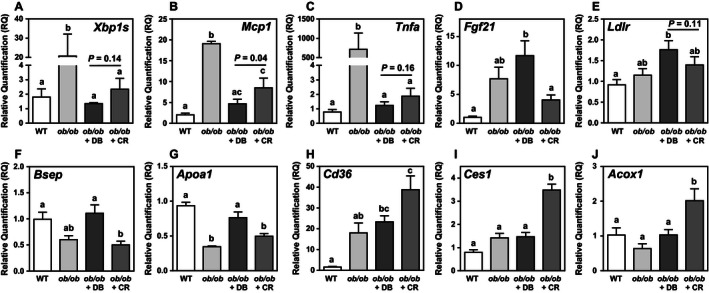
Didemnin B treatment alters the expression of some ER stress, and inflammatory and cholesterol metabolism genes. Relative mRNA contents of (A) *Xbp1s*, (B) *Mcp1*, (C) *Tnfa*, (D) *Fgf21*, (E) *Ldlr*, (F) *Bsep*, (G) *Apoa1*, (H) *Cd36*, (I) *Ces1*, and (J) *Acox1* were determined by qRT‐PCR. Data are means ± SEM,* n* = 8. Statistically significant differences were determined by ANOVA followed by post hoc tests comparing all groups. Bars with different letters are significantly different at *P* < 0.05, while those that share the same letter are not significantly different.

ER stress is known to be associated with hepatic lipotoxicity and disease progression from NAFLD to steatohepatitis in rodents (Pagliassotti 2012; Zhou and Liu 2014). Thus, we assessed markers of the UPR and ER stress response, and inflammation in all experimental groups by immunoblotting and multiplex quantitative PCR. No statistically significant differences in hepatic EEF1A1 and phosphorylated eIF2*α* protein were observed between groups by ANOVA (Fig. 3A, B). However, as we reported previously in leptin‐deficient mice, there were trends for increased EEF1A1 protein expression (Stoianov et al. 2015) and eIF2*α* phosphorylation (Ozcan et al. 2004) in *ob/ob* mice compared to lean control mice. Moreover, both didemnin B treatment and caloric restriction appeared to decrease EEF1A1 protein (Fig. 3A). Both GRP78 and phosphorylated JNK proteins were decreased by 53% and 38%, respectively, in didemnin B‐treated mice compared to *ob/ob* vehicle control mice (Fig. 3C, D), while caloric restriction only decreased phosphorylated JNK (Fig. 3D). At the transcript level, *Xbp1s*,* Mcp1*, and *Tnfa* were dramatically induced in obese mice, and we observed that these effects were reversed to a greater extent by didemnin B treatment than by caloric restriction alone (Fig. 4A–C). As expected, several other ER stress‐ and inflammation‐related transcripts were significantly induced in obese mice, but most were decreased to similar extents by didemnin B and caloric restriction (Table 2). Taken together, these data suggest that although didemnin B treatment did not inhibit the initiation of ER stress and induction of the UPR as indicated by eIF2*α* phosphorylation, it did diminish the later phases of the ER stress response (GRP78 and *Xbp1s* expression), and limited the activation of some inflammatory pathways (*Mcp1* and *Tnfa* expression) – both to a greater extent than caloric restriction alone.

### Didemnin B treatment normalizes plasma lipid profiles in obese mice

**Figure 5:**
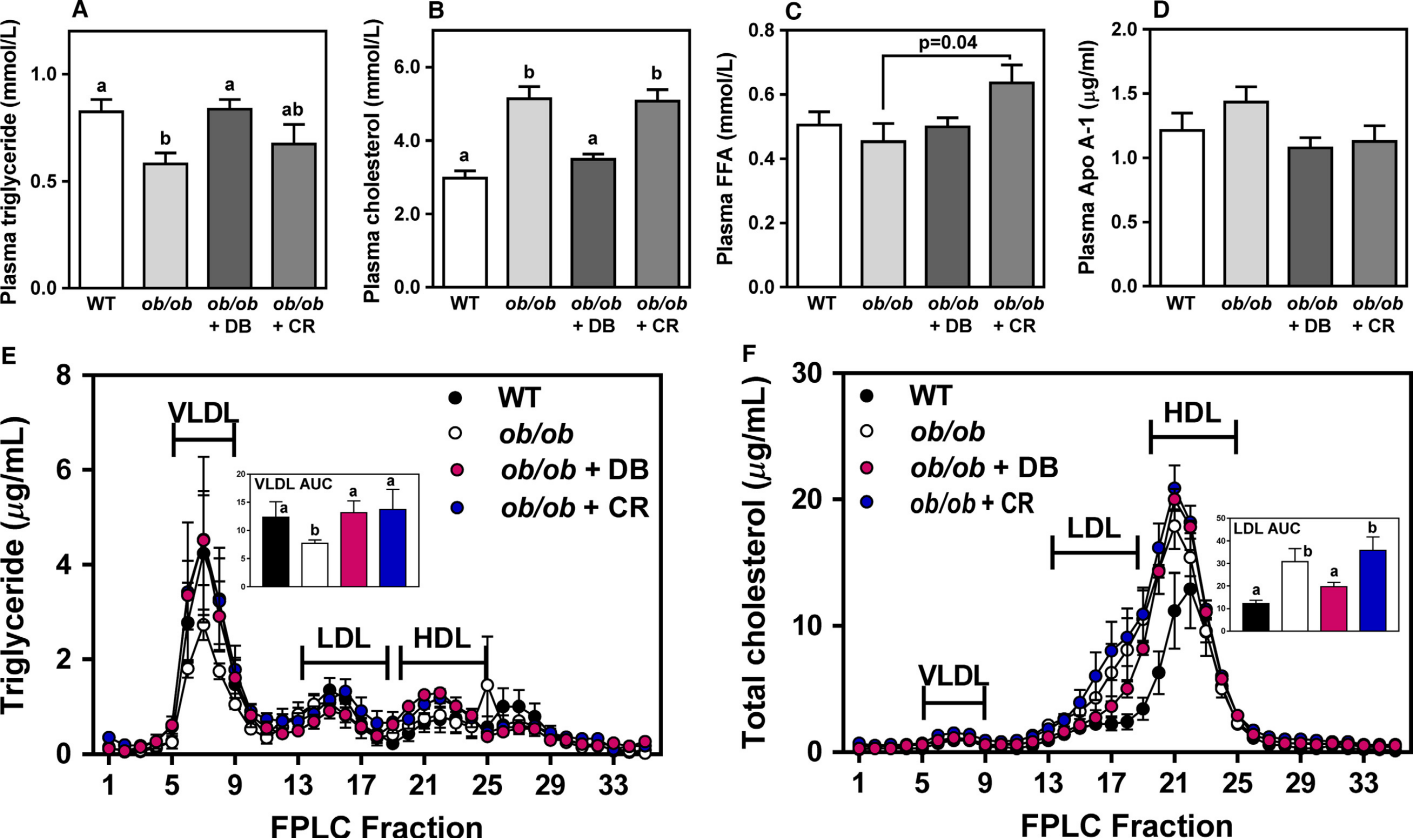
Didemnin B treatment improves plasma lipid and lipoprotein profiles. C57BL/6J (WT) and obese (*ob/ob*) mice were treated as described in Figure 1. (A) Plasma triglyceride, (B) cholesterol, and (C) free fatty acids (FFA) were measured using standard enzymatic, colorimetric assays. (D) Plasma apolipoprotein (apo) A‐1 levels were measured by ELISA. Data are means ± SEM,* n* = 8–12. (E) Triglyceride and (F) cholesterol concentrations were measured in very low‐density lipoprotein (VLDL), low‐density lipoprotein (LDL), and high‐density lipoprotein (HDL)‐eluted plasma fractions. Inset graphs show area under the curve (AUC) for VLDL triglyceride (fractions 5–10) and LDL cholesterol (fractions 13–19). Data are means ± SEM,* n* = 4. For all data, statistically significant differences were determined by ANOVA followed by post hoc tests comparing all groups. Bars with different letters are significantly different at *P* < 0.05, while those that share the same letter are not significantly different.

Severe hepatic steatosis and the progression of NAFLD is associated with impaired ability of the liver to export triglyceride‐rich lipoproteins (very low‐density lipoprotein [VLDL]) (Camus et al. 1988; Li et al. 1997; Wiegman et al. 2003; Coenen et al. 2007). Consistent with this early work, we found that plasma triglycerides were decreased in *ob/ob* mice compared to lean control mice (Fig. 5A). However, plasma triglycerides were returned to lean control levels in didemnin B‐treated and, to a lesser degree, in calorie‐restricted animals. Interestingly, plasma cholesterol levels were normalized in didemnin B‐treated mice, but not in calorie‐restricted mice (Fig. 5B). Plasma free fatty acid and apolipoprotein A‐1 levels were, for the most part, not significantly different between experimental groups (Fig. 5C, D), though increased free fatty acids were observed in calorie‐restricted mice compared to *ob/ob* control mice (Fig. 5C). Consistent with these plasma lipid data, plasma lipoprotein profiles for VLDL triglycerides (Fig. 5E, magenta symbols) and LDL cholesterol (Fig. 5F, magenta symbols) were normalized by didemnin B treatment, while caloric restriction only restored plasma VLDL triglycerides (Fig. 5E, blue symbols), and did not normalize LDL cholesterol (Fig. 5F, blue symbols). Calculated areas under the curve for VLDL triglyceride (Fig. 5E, inset) and LDL cholesterol (Fig. 5F, inset) provided further evidence of the correction of dyslipoproteinemia in mice treated with didemnin B.

Gene expression analyses provided some insight into the mechanisms underlying differences between the effects of didemnin B and caloric restriction on plasma lipids and lipoproteins. Didemnin B, but not caloric restriction, increased the expression of *Fgf21*,* Ldlr*, and *Bsep* (Fig. 4D–F), the gene products of which would be expected to decrease LDL in the circulation (Claudel et al. 2011; Gimeno and Moller 2014). Increased Ldlr expression could also account for the increased liver cholesteryl ester content we observed with didemnin B (Table 1), as incoming LDL cholesterol is re‐esterified following its release from lysosomes. Interestingly, *Apoa1* expression was also increased only by didemnin B (Fig. 4G), but was not mirrored by increased plasma ApoA1 (Fig. 5D), suggesting that high‐density lipoprotein clearance may be increased by didemnin B. Consistent with our observations of increased plasma free fatty acids with caloric restriction (Fig. 5C), we found that some genes associated with fasting, including *Cd36*,* Ces1*, and *Acox1*, were significantly induced in these mice, but not in didemnin B‐treated mice (Fig. 4H–J). These data suggest that, in contrast to calorie‐restricted *ob/ob* mice that typically gorge upon feeding, didemnin B‐treated mice were probably grazing ad libitum prior to the 6‐h fasting period.

**Table 2:** Gene expression analyses of liver tissues

Mouse strain	C57BL/6J	*ob/ob*	*ob/ob*	*ob/ob*
Treatment	Vehicle	Vehicle	Didemnin B	Calorie‐restricted vehicle
Lipid and mitochondrial metabolism				
*Cd36*	1.6 ± 0.3 (a)	18.0 ± 4.8 (ab)	23.4 ± 2.9 (bc)	38.8 ± 6.6 (c)
*Scd1*	1.0 ± 0.1	0.9 ± 0.2	1.6 ± 0.2	1.3 ± 0.2
*Ces1*	0.8 ± 0.1 (a)	1.4 ± 0.2 (a)	1.5 ± 0.2 (a)	3.5 ± 0.2 (b)
*Mogat1*	0.7 ± 0.2 (a)	50.8 ± 14.0 (ab)	92.2 ± 11.6 (b)	93.6 ± 16.5 (b)
*Mogat2*	0.8 ± 0.1 (a)	0.9 ± 0.2 (a)	1.5 ± 0.2 (ab)	1.8 ± 0.2 (b)
*Dgat1*	0.9 ± 0.1 (a)	0.8 ± 0.2 (a)	1.4 ± 0.2 (ab)	1.8 ± 0.2 (b)
*Dgat2*	1.0 ± 0.2	1.2 ± 0.3	1.7 ± 0.3	0.9 ± 0.1
*Agpat2*	0.9 ± 0.1 (a)	1.2 ± 0.2 (ab)	1.6 ± 0.2 (ab)	1.8 ± 0.2 (b)
*Agpat3*	1.0 ± 0.1 (a)	1.1 ± 0.2 (ab)	1.8 ± 0.2 (ab)	2.0 ± 0.3 (b)
*Lipin1*	0.6 ± 0.1	0.5 ± 0.1	0.8 ± 0.1	0.6 ± 0.1
*Lipin3*	1.0 ± 0.0 (a)	0.7 ± 0.2 (b)	1.2 ± 0.0 (a)	1.1 ± 0.0 (a)
*Cpt1a*	0.9 ± 0.1 (a)	1.1 ± 0.1 (ab)	1.7 ± 0.2 (bc)	1.9 ± 0.2 (c)
*Acadl*	1.0 ± 0.2	1.7 ± 0.7	1.4 ± 0.2	1.6 ± 0.2
*Acadvl*	0.9 ± 0.1	0.9 ± 1.3	1.2 ± 0.1	1.4 ± 0.2
*Acox1*	1.0 ± 0.2 (a)	0.6 ± 0.1 (a)	1.0 ± 0.2 (a)	2.0 ± 0.3 (b)
*Fabpi*	1.3 ± 0.2 (ab)	0.9 ± 0.2 (a)	1.7 ± 0.2 (b)	1.6 ± 0.2 (ab)
*Acly*	0.9 ± 0.1 (ab)	0.8 ± 0.2 (ab)	1.2 ± 0.3 (a)	0.4 ± 0.1 (b)
*Acaca*	1.0 ± 0.2	1.2 ± 0.4	2.0 ± 0.5	0.9 ± 0.2
*Praak1*	1.0 ± 0.1	1.0 ± 0.2	1.5 ± 0.2	1.6 ± 0.2
*Mlycd*	1.0 ± 0.1	1.0 ± 0.1	1.6 ± 0.2	1.5 ± 0.2
*Me1*	0.8 ± 0.1	1.5 ± 0.6	1.4 ± 0.3	1.0 ± 0.2
*Fasn*	1.0 ± 0.1 (ab)	1.3 ± 0.4 (ab)	2.1 ± 0.5 (a)	0.8 ± 0.2 (b)
*Plin2*	1.2 ± 0.2 (a)	2.1 ± 0.2 (ab)	2.4 ± 0.3 (b)	2.5 ± 0.4 (b)
*Elovl6*	1.1 ± 0.2 (ab)	2.1 ± 0.6 (ab)	2.9 ± 0.8 (a)	1.0 ± 0.2 (b)
*Ucp2*	1.0 ± 0.1 (a)	2.6 ± 0.6 (ab)	4.7 ± 0.8 (b)	4.0 ± 0.8 (b)
*Atp synthase*	1.1 ± 0.2	1.4 ± 0.3	1.2 ± 0.1	1.4 ± 0.2
*Sirt1*	1.1 ± 0.2	0.5 ± 0.1	0.9 ± 0.1	1.0 ± 0.1
*Sirt3*	1.0 ± 0.1	0.8 ± 0.1	1.8 ± 0.3	1.8 ± 0.3
Cholesterol and bile acid metabolism				
*Hmgcr*	1.5 ± 0.4	2.8 ± 1.7	1.7 ± 0.7	0.5 ± 0.1
*Abcg5*	0.9 ± 0.2 (a)	1.2 ± 0.1 (ab)	1.6 ± 0.2 (ab)	1.9 ± 0.2 (b)
*Abcg8*	0.8 ± 0.1	0.7 ± 0.1	1.0 ± 0.1	1.2 ± 0.1
*Soat1*	1.2 ± 0.2 (a)	5.7 ± 1.9 (b)	1.6 ± 0.2 (a)	1.4 ± 0.2 (a)
*Cyp7a1*	0.5 ± 0.1 (a)	0.4 ± 0.1 (a)	0.8 ± 0.1 (b)	0.6 ± 0.1 (ab)
*Cyp27a1*	2.3 ± 0.7	4.5 ± 1.8	2.1 ± 0.4	1.7 ± 0.2
*Cyp8b1*	1.0 ± 0.1	0.9 ± 0.1	1.1 ± 0.2	1.0 ± 0.2
*Bsep*	1.0 ± 0.1 (a)	0.6 ± 0.1 (ab)	1.1 ± 0.2 (a)	0.5 ± 0.1 (b)
*Ntcp*	0.9 ± 0.1	0.9 ± 0.2	1.0 ± 0.1	1.1 ± 0.2
Regulation of lipid and cholesterol metabolism				
*Scap*	0.9 ± 0.1	0.4 ± 0.1	1.0 ± 0.2	0.8 ± 0.1
*Insig1*	1.1 ± 0.1	0.8 ± 0.2	1.3 ± 0.3	0.6 ± 0.1
*Pparα*	0.8 ± 0.2	0.8 ± 0.2	1.2 ± 0.0	1.3 ± 0.1
*Nr1h3*	1.0 ± 0.1	0.9 ± 0.2	1.1 ± 0.1	1.2 ± 0.1
*Hr1h2*	0.9 ± 0.1	1.1 ± 0.3	1.0 ± 0.1	0.9 ± 0.1
*Srebf1*	0.8 ± 0.1	1.2 ± 0.3	1.3 ± 0.2	1.2 ± 0.2
*Srebf2*	0.9 ± 0.1	0.7 ± 0.1	0.9 ± 0.1	0.9 ± 0.1
*Shp*	1.0 ± 0.2	2.3 ± 1.0	1.7 ± 0.2	2.5 ± 0.6
*Insig2*	2.9 ± 0.9 (a)	69.9 ± 37.9 (b)	4.7 ± 0.6 (a)	6.7 ± 1.1 (a)
*Hnf1α*	1.0 ± 0.2	1.7 ± 0.9	0.8 ± 0.1	0.9 ± 0.1
*Fgf21*	1.0 ± 0.2 (a)	7.7 ± 2.0 (ab)	11.7 ± 2.6 (b)	4.0 ± 0.8 (a)
*S1p*	1.1 ± 0.1	1.5 ± 0.3	1.4 ± 0.1	1.4 ± 0.2
Lipoprotein metabolism				
*Ldlr*	0.9 ± 0.1 (a)	1.2 ± 0.2 (ab)	1.8 ± 0.2 (b)	1.4 ± 0.2 (ab)
*Mttp*	1.0 ± 0.1	0.8 ± 0.1	1.3 ± 0.2	1.2 ± 0.1
*Apob*	1.7 ± 0.5	31.1 ± 14.4	60.6 ± 26.2	27.9 ± 8.9
*Scarb1*	1.4 ± 0.5	0.7 ± 0.1	0.8 ± 0.1	0.8 ± 0.1
*Apoa1*	0.9 ± 0.0 (a)	0.3 ± 0.0 (b)	0.8 ± 0.1 (a)	0.5 ± 0.0 (b)
*Pcsk9*	1.0 ± 0.1	1.2 ± 0.9	0.5 ± 0.1	0.3 ± 0.1
Glucose metabolism				
*Pck1*	0.7 ± 0.1	1.0 ± 0.2	1.2 ± 0.1	1.4 ± 0.2
*G6pc*	0.7 ± 0.2	0.7 ± 0.2	0.7 ± 0.1	1.0 ± 0.2
*Pklr*	0.8 ± 0.1	2.7 ± 1.2	1.4 ± 0.2	1.0 ± 0.2
ER stress				
*Grp78*	1.3 ± 0.3	2.2 ± 0.9	1.3 ± 0.2	1.0 ± 0.2
*Atf4*	1.4 ± 0.2	1.8 ± 0.5	2.1 ± 0.3	1.2 ± 0.2
*Chop*	1.0 ± 0.0 (a)	2.6 ± 0.5 (b)	1.8 ± 0.1 (ab)	1.8 ± 0.3 (ab)
*Xbp1s*	1.8 ± 0.6 (a)	20.7 ± 11.4 (b)	1.4 ± 0.1 (a)	2.3 ± 0.8 (a)
Oxidative stress				
*Gss*	1.1 ± 0.2 (a)	2.7 ± 0.2 (b)	2.5 ± 0.2 (b)	2.5 ± 0.3 (b)
*Nox1*	1.2 ± 0.4 (a)	16.9 ± 4.2 (b)	6.1 ± 1.5 (ab)	6.0 ± 1.8 (ab)
*Gsr*	0.9 ± 0.1	2.9 ± 1.8	1.1 ± 0.1	1.5 ± 0.2
*Gpx1*	1.6 ± 0.4	2.4 ± 0.6	3.9 ± 1.0	2.3 ± 0.5
Inflammation				
*Cd68*	1.2 ± 0.2 (a)	2.1 ± 0.1 (b)	1.6 ± 0.2 (ab)	1.6 ± 0.3 (ab)
*Emr1*	1.5 ± 0.2 (a)	2.6 ± 0.4 (b)	1.8 ± 0.3 (ab)	1.4 ± 0.2 (a)
*Tnfα*	0.8 ± 0.2 (a)	713.6 ± 425.9 (b)	1.2 ± 0.2 (a)	1.9 ± 0.5 (a)
*Mcp1*	2.0 ± 0.4 (a)	19.1 ± 0.5 (b)	4.7 ± 1.1 (ac)	8.5 ± 2.3 (c)
*Tlr4*	2.3 ± 0.7	2.1 ± 0.7	2.3 ± 0.5	3.0 ± 0.6
*Pecam*	1.2 ± 0.2 (a)	2.1 ± 0.3 (b)	2.0 ± 0.3 (b)	2.0 ± 0.3 (b)
Fibrosis				
*Fn1*	0.9 ± 0.1 (a)	5.3 ± 1.4 (b)	3.3 ± 0.4 (bc)	2.0 ± 0.2 (ac)
*Fgf10*	0.6 ± 0.1	0.6 ± 0.3	0.7 ± 0.2	0.6 ± 0.1
*Vegfa*	1.4 ± 0.2	0.9 ± 0.3	1.1 ± 0.2	1.1 ± 0.3
*Wnt2b*	0.8 ± 0.2	3.5 ± 2.5	2.4 ± 0.7	1.1 ± 0.3

Five‐week‐old C57BL/6J and *ob/ob* mice were maintained ad libitum or pair‐fed (*ob/ob* + calorie restricted) on AIN‐76A semipurified diet for 4 weeks. During week 5, mice received i.p. injections of either vehicle or didemnin B (50 *μ*g/kg), as indicated, on days 1, 4, and 7. Relative mRNA contents of hepatic markers of lipid and mitochondrial metabolism, cholesterol and bile acid metabolism, regulation of lipid and cholesterol metabolism, lipoprotein metabolism, glucose metabolism, ER stress, oxidative stress, inflammation, and fibrosis were determined by multiplex qRT‐PCR. Data are means ± SEM, *n* = 8. Statistically significant differences were determined by ANOVA followed by post hoc tests comparing all groups. Values with different letters are significantly different at *P* < 0.05, while those that share the same letter are not significantly different.

### Didemnin B treatment improves glucose homeostasis in obese mice primarily through decreased food intake

**Figure 6:**
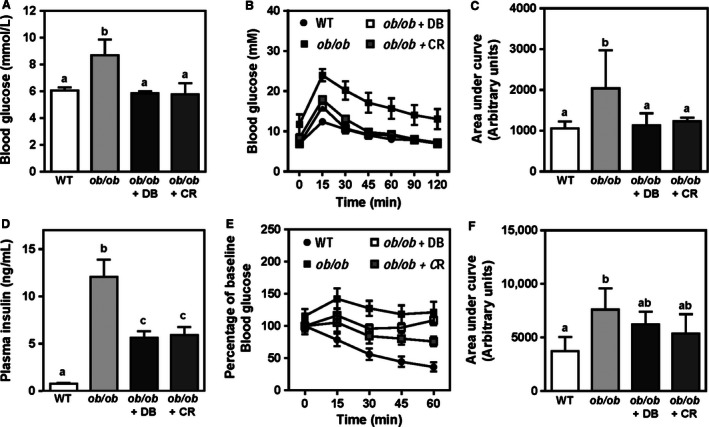
Both didemnin B treatment and caloric restriction improve glucose homeostasis. C57BL/6J (WT) and obese (*ob/ob*) mice were treated as described in Figure 1. (A) Prior to sacrifice, fasting blood glucose levels were measured by hand‐held glucometer. (B) Glucose tolerance was measured following a 6‐h fast and oral gavage of 1 g glucose/kg body weight. (C) Areas under the curve were calculated from data in B. (D) Plasma insulin levels were measured by ELISA. (E) Insulin tolerance was measured following a 6‐h fast and i.p. injection of 0.6 IU insulin/kg body weight. (F) Areas under the curve were calculated from data in E. Data are means ± SEM,* n* = 8–12. Statistically significant differences were determined by ANOVA followed by post hoc tests comparing all groups. Bars with different letters are significantly different at *P* < 0.05, while those that share the same letter are not significantly different.

Hepatic lipotoxicity is thought to contribute to insulin resistance and disrupted glucose homeostasis during NAFLD (Tessari et al. 2009). Therefore, parameters of glucose homeostasis were measured in all experimental groups. Fasting blood glucose and oral glucose tolerance were improved to levels observed in wild‐type mice, in both didemnin B and calorie‐restricted animals (Fig. 6A–C). Similarly, fasting plasma insulin and insulin tolerance were improved in both didemnin B and calorie‐restricted mice (Fig. 6D–F). These data suggest that improvements in glucose homeostasis observed in mice treated with didemnin B were secondary to decreased food consumption. Expression analyses of genes involved in glucose metabolism further supported this conclusion (Table 2).

**Figure 7:**
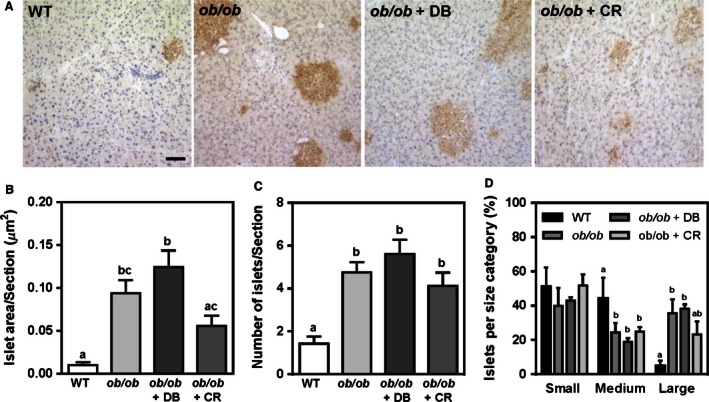
Acute treatment with didemnin B does not alter pancreatic islet morphology. C57BL/6J (WT) and obese (*ob/ob*) mice were treated as described in Figure 1. (A) Pancreatic tissues were harvested, embedded in optimal cutting temperature, sectioned, and stained for insulin. Scale bar represents 100 *μ*m. (B) Total islet areas and (C) islet densities were assessed in serial sections per mouse. (D) Pancreatic islets were categorized as small (<2500 *μ*m^2^), medium (2500 *μ*m^2^ ≤ *x* ≤ 10,000 *μ*m^2^), or large (>10,000 *μ*m^2^) to determine islet size distribution in serial sections across different treatment groups. Data are means ± SEM,* n* = 8–12. Statistically significant differences were determined by ANOVA followed by post hoc tests comparing all groups. Bars with different letters are significantly different at *P* < 0.05, while those that share the same letter are not significantly different.

Since didemnin B is known to accumulate in the pancreas after i.p. administration (Beasley et al. 2005), we examined gross pancreatic and islet morphology by insulin staining and light microscopy (Fig. 7A). As expected, total islet areas and numbers of islets were significantly increased in *ob/ob* compared to lean mice (Fig. 7B, C). Total islet areas were unaffected by didemnin B treatment, but showed a trend toward being decreased in calorie‐restricted mice (Fig. 7B). Consistent with the effect of caloric restriction on total islet areas, there was a trend for decreased large islets (>10,000 *μ*m^2^) in these mice (Fig. 7D). In contrast, islet size distributions were not significantly different in didemnin B‐treated mice compared to *ob/ob* mice (Fig. 7D). These data suggest that acute treatment with didemnin B did not significantly alter pancreas or pancreatic islet morphology, despite the stimulus for decreased *β* cell mass stemming from caloric restriction.

## Discussion

Despite the prevalence of NAFLD, few treatment options exist to prevent its progression. Prolonged hepatic ER stress is recognized as a contributing factor to progression from relatively benign steatosis to steatohepatitis, as ER stress response pathways can induce the expression of inflammatory and proapoptotic factors in hepatocytes (Lee and Ozcan 2014). We and others have proposed that inhibiting the restoration of protein synthesis that occurs late in the ER stress response could prevent a deleterious anabolic response (Han et al. 2013; Krokowski et al. 2013; Stoianov et al. 2015). Here, we showed that acute treatment of obese mice with the EEF1A inhibitor, didemnin B, modestly decreased food intake, and improved biochemical, histological, and molecular features of NAFLD. Didemnin B‐specific effects, that is, those that could not completely be accounted for by decreased caloric intake, included reductions in liver triglycerides, hepatic markers of ER stress, hepatic lobular inflammation, plasma triglycerides, and plasma total and LDL cholesterol – all indicating decreased hepatic lipotoxicity and improved liver function.

The decreased food intake we observed in leptin‐deficient mice treated with didemnin B was unexpected and intriguing. These animals showed no evidence of overt illness, distress, or liver toxicity, and limited their food intake to levels equivalent to wild‐type mice. Based on the lack of increase in plasma free fatty acids and genes typically induced in mouse liver as an early response to fasting (i.e., within 24 h) (Sokolovic et al. 2008), it is possible that didemnin B‐treated mice were grazing in accordance with their decreased appetites before the 6‐h fast that preceded sacrifice. It is likely that, for the course of the study, these mice experienced chronic calorie reduction, but were feeding ad libitum. In contrast, the calorie‐restricted *ob/ob* group likely consumed most food at the time it was provided, as is typical of this hyperphagic strain, and subsequently underwent a longer, but acute, fast (approximately 20 h) prior to sacrifice. Thus, for the course of the study, these mice likely experienced intermittent, acute fasting. Didemnin B is not known to accumulate significantly in brain tissue of mice after i.p. injection (Beasley et al. 2005), making it unlikely that our treatment activated central satiety pathways. However, didemnin B accumulates at relatively high concentrations in the lower gastrointestinal tract and pancreas after i.p. delivery (Beasley et al. 2005). Both the intestine and pancreas are known sites of production of the orexigenic peptide, ghrelin, with the intestine being the more important source in mice (Wierup et al. 2007, 2014). The possibility that didemnin B inhibited intestinal ghrelin synthesis resulting in appetite suppression in leptin‐deficient mice, and feeding behaviors in didemnin B‐treated animals in general, warrants further investigation.

**Figure 8:**
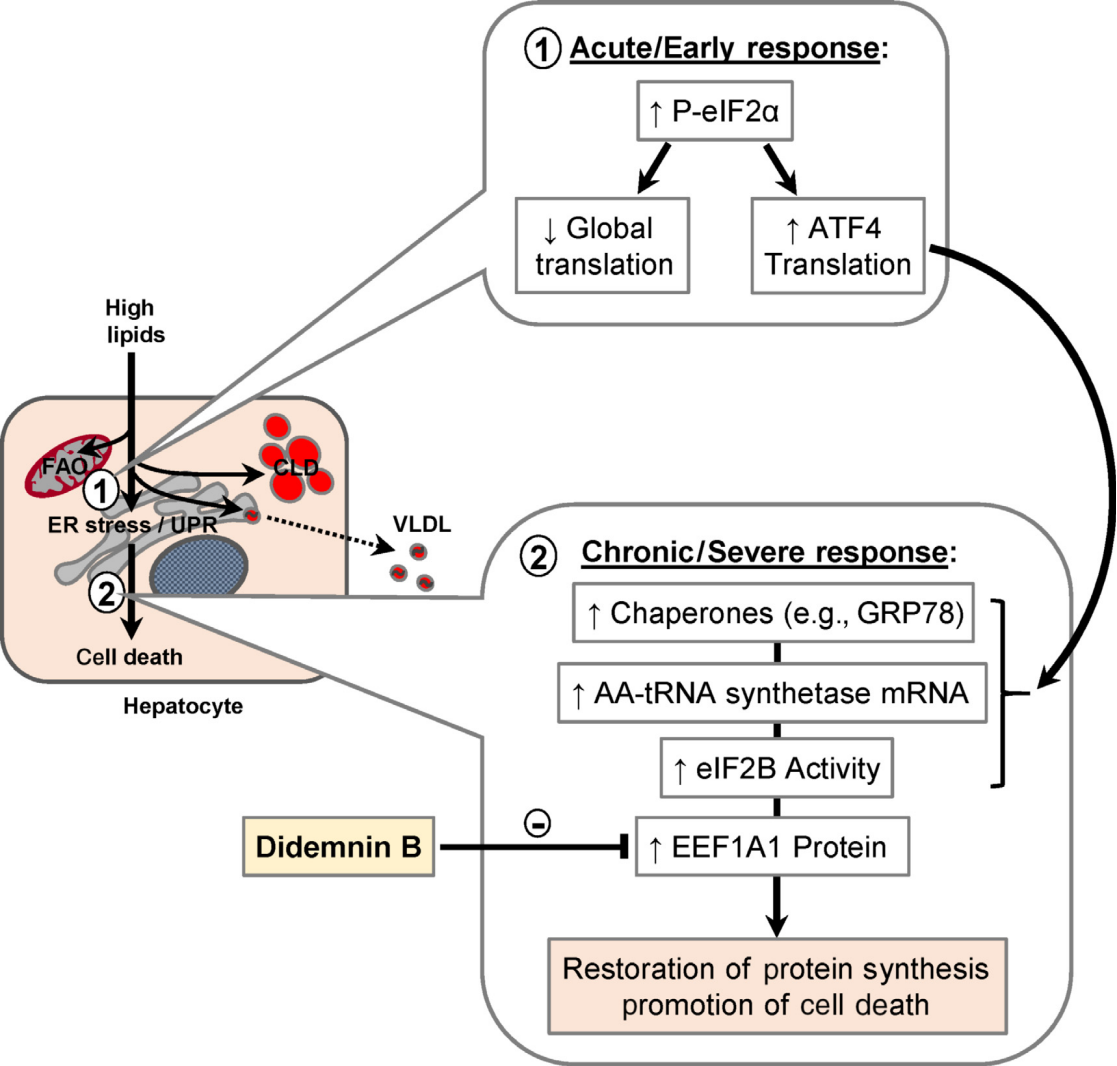
Working model for the potential beneficial effects of EEF1A1 inhibition during lipid‐induced hepatic ER stress. ER stress occurs in hepatocytes as the exogenous delivery and/or endogenous synthesis of fatty acids exceeds their ability to oxidize them (FAO, fatty acid oxidation), to store them safely as triglycerides (CLD, cytosolic lipid droplets), or to export them into plasma (VLDL, very low‐density lipoprotein). The initial, acute, or early response to ER stress (1) involves phosphorylation of eIF2a (P‐eIF2a) to attenuate global protein translation, and the expression of transcription factors (e.g., ATF4) to induce chaperones, in order to reduce the burden of unfolded protein within the ER (unfolded protein response, UPR). However, with chronic or severe stress (2), protein synthesis rates recover through a multifaceted, ATF4‐dependent mechanism, which re‐establishes the anabolic burden at the ER, leading to cell death. In hepatocytes, EEF1A1 is highly localized to the ER, is induced in response to fatty acid excess and ER stress, and promotes cell death under these conditions. Partially blocking protein synthesis through inhibition of EEF1A1, with a compound such as didemnin B, could limit the deleterious anabolic response associated with prolonged or severe ER stress.

Didemnin B accumulates to the greatest extent in liver after i.p. administration, and exhibits a relatively long half‐life of 72 h in this tissue (Beasley et al. 2005). We took advantage of these characteristics to attempt to target liver EEF1A1 activity, and limit the damaging aspects of the hepatic ER stress response in obese mice with NAFLD. We did not observe decreased steady‐state total liver protein or albumin content in didemnin B‐treated mice, which is consistent with the partial inhibition of elongation activity predicted for this dose (Robert et al. 2009). Consistent with our recent studies in HepG2 human hepatocyte‐like cells (Stoianov et al. 2015), didemnin B treatment did not inhibit initiation of ER stress and induction of the UPR as indicated by eIF2*α* phosphorylation, but did diminish the later phases of the ER stress response (GRP78 and *Xbp1s* expression). This was associated with decreased activation of some inflammatory pathways (*Mcp1* and *Tnfa* expression), and decreased lobular inflammation, to a greater extent than caloric restriction alone. Although we could not directly measure acute hepatic protein synthesis in this study, these data combined with our previous work in HepG2 cells (Stoianov et al. 2015), and the known accumulation of didemnin B in liver (Beasley et al. 2005), potentially support the concept that inhibiting protein synthesis may be beneficial for treating ER stress‐related diseases (Han et al. 2013) (Fig. 8). We cannot absolutely exclude the possibility that didemnin B also inhibited lysosomal palmitoyl‐protein thioesterase 1 (PPT1) (the only other known didemnin B target) in our studies (Meng et al. 1998; Potts et al. 2015). However, unlike EEF1A1 in liver, PPT1 is thought to be dispensable for general cell viability and function, but has a predominant role in maintaining neuronal function (Gupta et al. 2001).

To further investigate liver function of didemnin B‐treated mice, we measured circulating lipids and lipoproteins as an indicator of hepatic lipid metabolism and transport. Consistent with improved ER function, didemnin B treatment restored plasma triglyceride and VLDL concentrations to those of lean control mice. We speculate that by limiting protein synthesis at the ER (by inhibition of EEF1A1), nascent VLDL particles were afforded the resources required for complete assembly and secretion rather than being targeted to degradation in response to ER stress (Ota et al. 2008). Interestingly, we found that didemnin B also normalized both plasma total cholesterol and LDL cholesterol. In light of restored circulating VLDL concentrations, we reasoned that this was likely a result of increased LDL clearance. Our observations that didemnin B treatment tended to increase liver cholesteryl ester content, *Fgf21*,* Ldlr*, and *Bsep* expression support this possibility. FGF21 is thought to increase *Ldlr* expression in hepatocytes (Li et al. 2012), while *Bsep* expression is predominantly regulated by a feed‐forward mechanism dependent on hepatocyte bile acid content (Claudel et al. 2011). Chronic fasting and ketogenic diets are potent inducers of FGF21 production (Gimeno and Moller 2014). Thus, we think it possible that the combination of decreased appetite and inhibition of protein synthesis in didemnin B‐treated mice mimicked long‐term caloric restriction, or adaptive starvation, more effectively than the intermittent, acute fasting experienced by vehicle‐treated, calorie‐restricted *ob/ob* mice. This in turn resulted in greater upregulation of *Fgf21* expression in didemnin B‐treated mice. Whether FGF21 is required for the effects of didemnin B on plasma lipids and lipoproteins may be an interesting avenue for further study.

To our knowledge, this is the first report of the potential benefit of inhibition of EEF1A1, or chemical inhibition of peptide elongation, in an in vivo model of ER stress‐related disease such as NAFLD. Of note, a new EEF1A inhibitor, nannocystin A, has recently been isolated, which shares overlap in its EEF1A binding site with didemnin B (Krastel et al. 2015), providing an additional tool for further study and development of potential therapeutics targeting protein synthesis through EEF1A1. Future investigations in animal models of progressive NAFLD and liver fibrosis, and in the cell types involved in hepatic inflammation and fibrosis, will be critical for determining whether didemnin B, or inhibition of EEF1A1, will be effective in later stages of this disease.

## Conflict of Interest

The authors have no conflicts of interest, financial or otherwise, to disclose.
